# Projecting future health and service use among older people in Ireland: an overview of a dynamic microsimulation model in The Irish Longitudinal Study on Ageing (TILDA)

**DOI:** 10.12688/hrbopenres.13525.1

**Published:** 2022-03-21

**Authors:** Peter May, Charles Normand, Soraya Matthews, Rose Anne Kenny, Roman Romero-Ortuno, Bryan Tysinger

**Affiliations:** 1 Centre for Health Policy and Management, Trinity College Dublin, 3-4 Foster Place, Dublin, D2, Ireland; 2 The Irish Longitudinal Study on Ageing, Trinity College Dublin, Pearse Street, Dublin, D2, Ireland; 3 Cicely Saunders Institute, King's College London, Denmark Hill, London, SE1 1UL, UK; 4 Global Brain Health Institute, Trinity College Dublin, Lloyd Institute, Dublin, D2, Ireland; 5 Leonard D. Schaeffer Center for Health Policy and Economics, University of Southern California, Los Angeles, CA, 90007, USA

**Keywords:** ageing, mortality, health, health care use, projection, microsimulation, policy

## Abstract

**Background:** Demographic ageing is a population health success story but poses unprecedented policy challenges in the 21st century. Policymakers must prepare health systems, economies and societies for these challenges. Policy choices can be usefully informed by models that evaluate outcomes and trade-offs in advance under different scenarios.

**Methods: **We developed a dynamic demographic-economic microsimulation model for the population aged 50 and over in Ireland: the Irish Future Older Adults Model (IFOAM).  Our principal dataset was The Irish Longitudinal Study on Ageing (TILDA). We employed first-order Markovian competing risks models to estimate transition probabilities of TILDA participants to different outcomes: diagnosis of serious diseases, functional limitations, risk-modifying behaviours, health care use and mortality. We combined transition probabilities with the characteristics of the stock population to estimate biennial changes in outcome state.
***  ***

**Results: **IFOAM projections estimated large annual increases in total deaths, in the number of people living and dying with serious illness and functional impairment, and in demand for hospital care between 2018 and 2040.  The most important driver of these increases is the rising absolute number of older people in Ireland as the population ages. The increasing proportion of older old and oldest old citizens is projected to increase the average prevalence of chronic conditions and functional limitations. We deemed internal validity to be good but lacked external benchmarks for validation and corroboration of most outcomes.

**Conclusion: **We have developed and validated a microsimulation model that predicts future health and related outcomes among older people in Ireland.  Future research should address identified policy questions. The model enhances the capacity of researchers and policymakers to quantitatively forecast future health and economic dynamics among older people in Ireland, to evaluate ex ante policy responses to these dynamics, and to collaborate internationally on global challenges associated with demographic ageing.

## Introduction

### Background

Demographic ageing is a population health success story but poses unprecedented policy challenges in the 21st century. The squaring of the age pyramid, first in high-income countries and then the rest of the world, is affecting every facet of society ^1^. There are increasing pressures on the economy and on health care, and on the interaction between the two ^2^. Health and social care systems face large increases in the number of people living and dying with multiple serious chronic conditions ^3^. It is projected that there will be relatively fewer people of working age to support health care and pensions through taxation ^1^, growing scarcity of health professionals to provide the required services ^2^, and increasing pressure for family members to supplement the formal system with unpaid support ^4^. Furthermore, the economic and ill-health burdens are more likely to fall on the most disadvantaged ^5, 6^.

Governments must weigh different policies to maximise population health and economic opportunities associated with population ageing. For example, investment in public health interventions may avoid or delay the onset of chronic diseases, improving population health, and yielding cost-savings for health care systems ^7^; extending the retirement age may mitigate projected welfare losses associated with demographic ageing ^8^. These policy choices are complex, entailing measurements and trade-offs across multiple, often inter-related, domains including disease prevalence, health care use, life expectancy, quality of life, public spending, household wealth and individual preferences ^2^. The relative importance of these domains may change as the population ages, for example specific conditions such as dementia and frailty will play an increasing role in policy ^9^, and the uneven distribution of effects across different social groups is also a substantial concern ^6, 10^.

### Rationale and context

Ireland is early in the population ageing process relative to other high-income countries ^11^, but faces the same structural challenges as other nations. The Irish Government must best prepare the health system and economy for these challenges. Policy choices can be usefully informed by models that evaluate outcomes and trade-offs under different scenarios.

In this paper we introduce a model that can support decision-making for older populations in Ireland. We follow an approach pioneered in the United States with the Future Elderly Model (FEM) ^12^. The FEM is a microsimulation model – that is, it projects future health and economic outcomes using individual-level data. Specifically, it draws on the biennial Health and Retirement Survey (HRS) to use demographic factors (age, sex, ethnicity), socioeconomic factors (education, past employment), health status (chronic disease diagnoses, functional limitations) and risk-modifying behaviours (smoking, drinking, exercise) to predict future health outcomes (diagnoses, functionality, mortality) and economic outcomes (health care use, income, assets, pensions) ^13^.

By disaggregating at the individual level the complex inter-play between these factors, the FEM estimates future health (e.g. incidence of hypertension) and associated economic (e.g. missed employment) outcomes among Americans aged 50+. More importantly, it can estimate how changes to policy or individual health may affect these future outcomes – for example, does reducing incidence of hypertension delay dementia onset? ^14^ – and so help policymakers to prioritise effective interventions and efficient resource allocation. The HRS is part of an international family of studies of ageing ^15^. Family-member studies have developed FEM adaptations in Japan ^16^, Singapore ^17^, Korea ^18^, Mexico ^19^, and elsewhere ^20, 21^.

### Aim

We built a dynamic demographic-economic microsimulation model for the Irish population aged 50 and over. We call this the Irish Future Older Adults Model (IFOAM). Our principal dataset was The Irish Longitudinal Study on Ageing (TILDA) ^22^, the Irish member of the HRS family.

The aim of this paper is to document the methods used in the IFOAM, to validate its predictive accuracy against internal and external benchmarks, and to consider potential applications in projecting and supporting future older populations in Ireland.

## Methods

### Setting and context

Ireland is a country of approximately five million people in north-western Europe. The population is younger than the European Union average but faces the same basic demographic pressures as all high-income countries: increasing longevity, falling fertility, rising health and social care expenditures, and a growing workforce dependency ratio ^23, 24^. A younger population translates to fast-growing population health need; for example, the number of people dying with an incurable illness is projected to increase approximately 90% in Ireland over the next 30 years, compared to an increase of approximately 30% in England and Wales, and Scotland ^11, 25, 26^.

**Figure 1.**
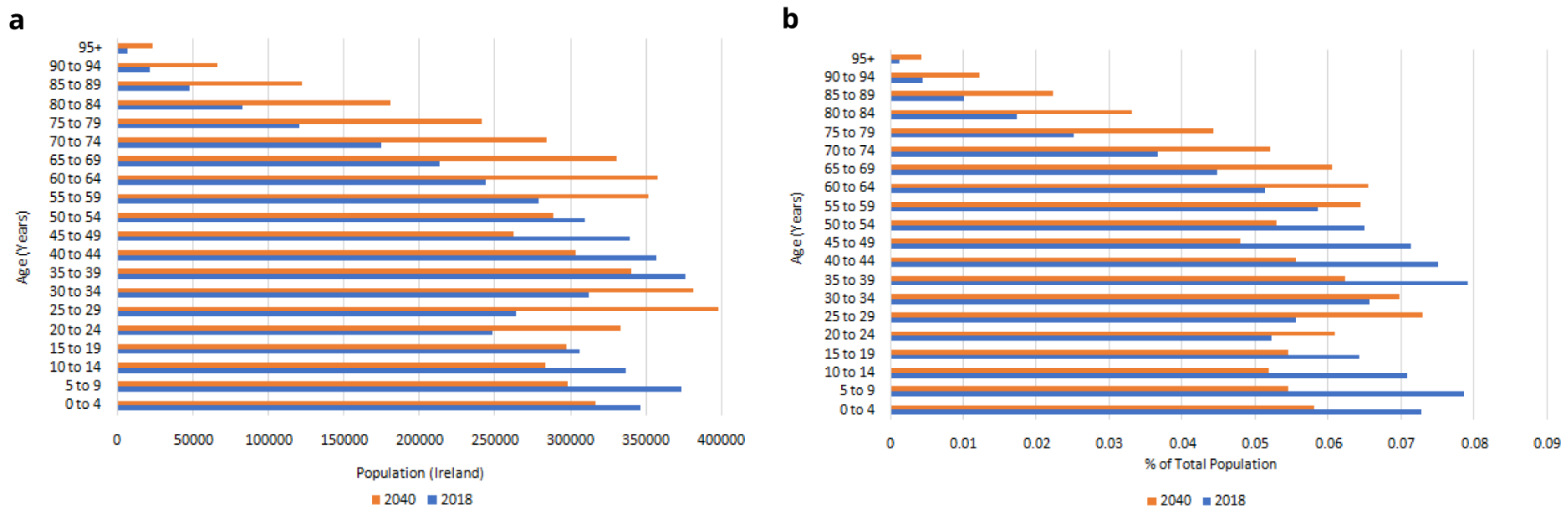
**a**. Irish population, 2018–2040. **b**. Age distribution, 2018-2040. Source: Central Statistics Office ^27^.

Trends in the Irish population are illustrated in Figure 1. Official statistics project that the total population will increase from 4,759,597 in 2018 to 5,460,552 in 2040, with significant increases in all age bands over 55 years ( Figure 1a) ^27^. When these age bands are expressed as a proportion of the population, there will be relatively fewer people aged under 55 in 2040 than in 2018, and relatively more people aged over 60, with the biggest relative increases in the oldest age groups ( Figure 1b). There are therefore two important dynamics at play in Ireland’s population-level ageing: the total number of older people is growing rapidly; and their age distribution is shifting upwards, meaning that the larger older populations aged 50+ in the next two decades will on average be older than today, with – all else held constant - higher prevalence of serious disease, functional limitations and other population health risks.

In Ireland, there is mixed public/private provision of health care. A means-tested medical card confers free hospital and general practitioner (GP) care, and subsidises pharmaceutical prescriptions. A means-tested GP card confers free GP care using wider eligibility criteria than the medical card, and all people aged over 70 have qualified automatically for a GP card since 2015 ^28^. Those without a medical card pay out-of-pocket co-payments for hospital visits and pharmaceuticals, and all primary care costs. Voluntary private insurance affords faster access to some treatments and procedures. International comparisons show that primary care capacity is low and reliance on acute inpatient care high, which contributes to high per-capita spending on health compared to neighbouring countries ^29^.

### Data

Our primary data source was The Irish Longitudinal Study on Ageing (TILDA), a biennial study of adults in Ireland. At Wave 1 in 2009-2010, a population-representative sample of 8,174 community-dwelling people aged 50+ were enrolled ^30^. Full details of the TILDA study design, recruitment, consent and data collection are available elsewhere ^22^. Briefly, data were collected using a computer-assisted personal interview (CAPI) and a self-completion questionnaire (SCQ) on demographic and socioeconomic characteristics such as marital status and history, education, labour market status, income and asset levels, as well as detailed information on healthcare utilisation, physical health, mental health and cognitive function. Qualified nurses conducted an assessment of objective health measures, both at home (at each wave) and in greater detail at a dedicated health assessment centre (at waves 1 and 3). Ethical approval for each wave was obtained from the Faculty of Health Sciences Research Ethics Committee in Trinity College Dublin. Participants were provided with sufficient information to make an informed decision about their participation including advance notice of the study; participants were free to refuse to take part in any of the parts or withdraw at any time without justification; for each CAPI question, possible answers included “Refuse to answer” and “Don’t know”. 

The recruited sample were invited to participate in CAPI and SCQ follow-up at Wave 2 (2012), Wave 3 (2014), Wave 4 (2016) and Wave 5 (2018). Wave 6, also including a health centre assessment, was planned for 2020 but delayed to 2021 due to the COVID-19 pandemic. The version of the model that we report in this paper therefore uses the first five waves of data. An additional 330 participants were recruited aged under 50 but a spouse of a participant aged over 50; where those participants were older than 50 in later waves we included them in the model too. 

Secondary data sources were the Central Statistics Office (CSO) census data, for the purposes of population weighting and mortality adjustment, and the General Register Office (GRO) to identify deaths ^31^. All deaths in Ireland are recorded with the GRO and the GRO is linked to TILDA in a process detailed elsewhere ^32^.

### Variables

***Independent variables.*** All predictors used in the model were taken or adapted from the TILDA CAPI conducted face-to-face with participants during each of the first five waves. These predictors are summarised in Table 1. We used age, sex, education level, diagnosis of specific serious illnesses, functional limitations, body mass index (BMI) and smoking history.

**Table 1.** Overview of variables.

Variable	Definition
*Sociodemographics*	
Age	Years
Sex	Male | Female
Education: Highest achieved	Primary, Secondary, Tertiary
*Risk factors*	
BMI	Weight in kilograms/(Height in metres^2)
In the last two years, have you stopped smoking?	Yes | No
*Diagnoses*	
Has a doctor told you that you have the following conditions [ *Diabetes/* * Cancer/ Lung disease/ Hypertension/ Heart disease/ Stroke*]?	Yes | No
*Functional limitations*	
ADLs: Because of a health or memory problem, do you have difficulty doing any of the following activities: dressing bathing, eating?	Total difficulties (/3): 0 | 1 | 2 | 3
IADLs: Because of a health or memory problem, do you have difficulty doing any of the following activities: preparing a hot meal, shopping for groceries, making telephone calls, taking medications, managing money?	Total difficulties (/5): 0 | 1 | 2+
*Mortality*	
Mortality	Confirmed died via GRO or family member
*Health care utilisation in prior 12 months*	
ED visits	Count
Inpatient admissions	Count
Outpatient visits	Count

**Legend:** (I)ADLs: (Instrumental) Activities of Daily Living. BMI: Body mass index. ED: emergency department. **Sources:** All variables were self-reported in the TILDA CAPI except for BMI, which was measured by a nurse in a health assessment centre at Wave 1 and self-reported in all subsequent waves. This created an inconsistency problem, where Wave 1 BMI was higher than at later Waves for the majority of participants. For data consistency we used BMI as an outcome in Wave 2 onwards only, and we used BMI as a predictor in all waves after adjusting the Wave 1 data to match the distribution of later Waves (e.g. if a participant had BMI in the 60 ^th^ percentile in Wave 1 then we imputed their BMI as the 60 ^th^ percentile value for later waves). **Notes:** Each diagnosis question was asked individually except for heart disease, which combines diagnosis of any one of heart attack, congestive heart failure, angina and cardiac arrhythmia. TILDA asked about six ADLs and six IADLs, but the current version of the model runs on the publicly available harmonised dataset, which contains only three and five of these respectively. Future iterations of the model will use the full dataset and model all six difficulties in each index. ADLs were used from Wave 2 onwards because of a measurement inconsistency: Wave 1 CAPI asked, *“Because of a health or memory problem, do you have difficulty [dressing, including putting on shoes and socks]?”* Wave 2 onwards asked, *“Because of a health or memory problem, do you have difficulty [dressing]?”*

***Dependent variables.*** We modelled five types of outcome: risk factors, diagnoses, functional limitations, mortality and health care use. These outcomes are summarised in Table 1. Mortality was identified through GRO linkage and in some instances a family member reported a participant’s death to TILDA directly. Disease diagnoses were modelled as irreversible (‘absorbing’) states (e.g. ‘have you ever had a diagnosis of cancer?’). Risk factors, health status and health care use variables were self-reported in the TILDA CAPI. 

### Model design and statistical methods

***Model structure.*** The IFOAM uses three population files compiled using TILDA data: stock population, transitions population and replenishing population. The stock population was the starting point for the simulation; we used the TILDA sample at baseline in 2010. The transitions population contains all participants from Wave 2 onwards and was used to estimate the two-year probabilities of transition for different outcomes conditional on individuals’ characteristics. These models were used for simulating the future outcomes of individuals in IFOAM. The replenishing population is a synthesised group of 50-51-year-olds, introduced to keep the simulation population nationally-representative as the simulation progressed.

**Figure 2.**
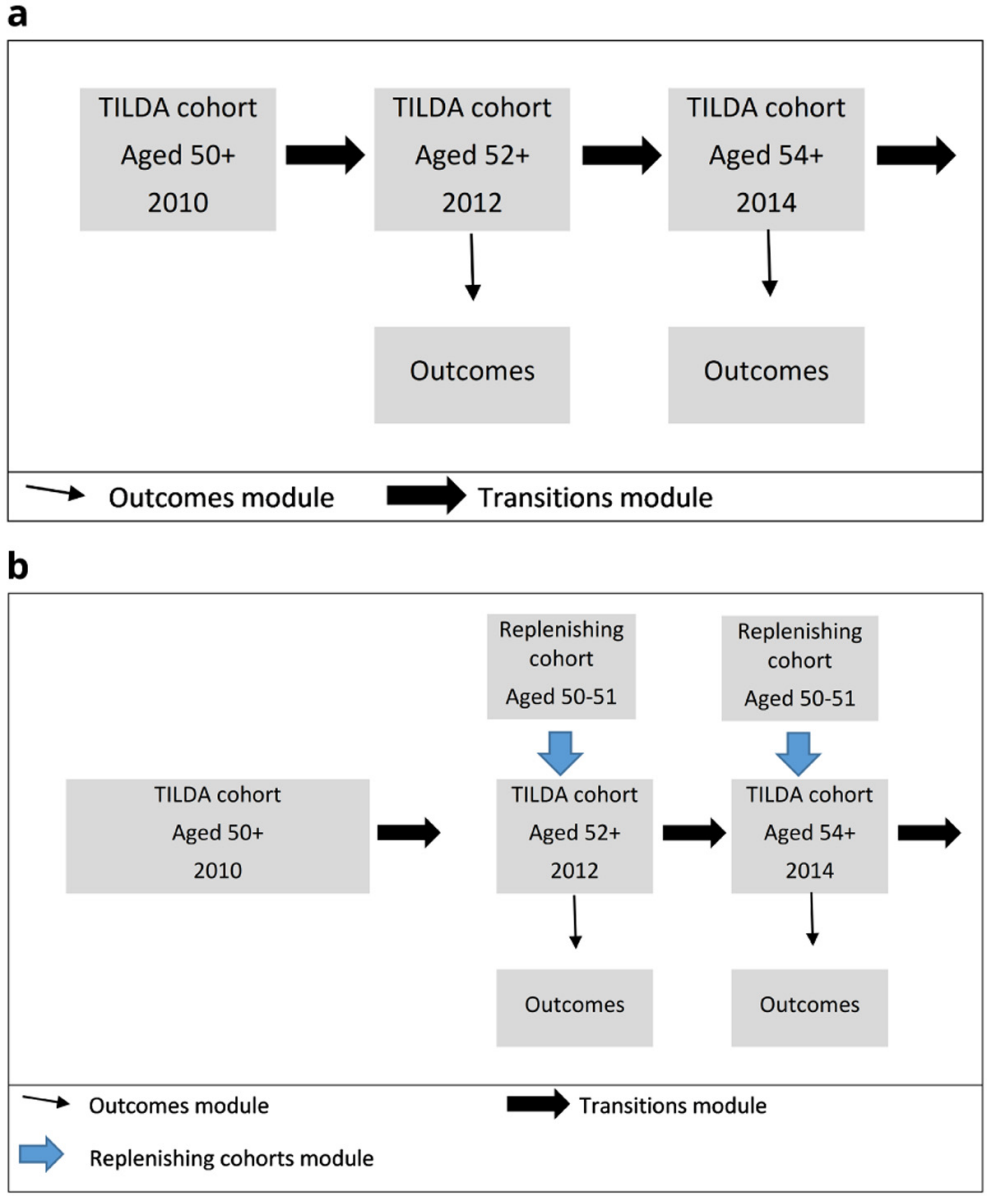
**a**. Cohort Simulation **b**. Population Simulation.

This structure is illustrated in Figure 2, which shows the two different types of simulation. Figure 2a illustrates a cohort simulation. We combined transition probabilities with the characteristics of the stock population to estimate biennial changes in outcome state: mortality, diagnoses, functional limitations, risk factors and health care use. Figure 2b illustrates a population simulation. As well as estimating biennial outcomes in the stock population, the sample was replenished with 50-to-51-year-olds every two years to preserve population representativeness.

***Statistical methods.*** We employed first-order Markovian competing risks models to estimate transitions, which means that observations can be at risk of more than one mutually exclusive event; e.g. in modelling two-year incidence of cancer or health care use in older people, it is essential to take into account mortality risk as a competing risk since dying precludes any other outcomes from subsequently occurring ^33, 34^. Transition models are summarised in Table 2. Outcomes were modelled using predictors from the previous wave, and we choose predictors for outcomes based on the following conceptual causal order: risk factors -> chronic disease -> functional limitations -> mortality/health care use.

**Table 2.** Overview of transition models.

Outcome	Outcome variable	Model type	Predictors
*Risk factors*			
BMI	Continuous	OLS	Age, sex, education, BMI
Stop smoking	Binary; reversible	Probit	Age, sex, education
*Diagnoses incidence*			
Diabetes	Binary; absorbing state	Probit	Age, sex, education, BMI
Cancer	Binary; absorbing state	Probit	Age, sex, education, current smoker, past smoker
Lung disease	Binary; absorbing state	Probit	Age, sex, education, current smoker, past smoker
Hypertension	Binary; absorbing state	Probit	Age, sex, education, diabetes
Heart disease	Binary; absorbing state	Probit	Age, sex, education, diabetes, hypertension, BMI
Stroke	Binary; absorbing state	Probit	Age, sex, education, diabetes, hypertension, cancer, heart disease
*Functional limitations*			
ADLs	Ordered	Ordered probit	Age, sex, education, diabetes, hypertension, cancer, heart disease, lung disease, stroke, ADLs, IADLs
IADLs	Ordered	Ordered probit	
*Mortality incidence*			
Mortality	Binary; absorbing state	Probit	Age, sex, education, diabetes, hypertension, cancer, heart disease, lung disease, stroke, ADLs, IADLs
*Health care utilisation*			
ED visits	Count	2-part; Poisson	Age, sex, education, diabetes, hypertension, cancer, heart disease, lung disease, stroke, ADLs, IADLs, prior use of the outcome variable
Inpatient admissions	Count	2-part; Poisson	
Outpatient visits	Count	2-part; Poisson	

**Legend:** (I)ADLs: (Instrumental) Activities of Daily Living. BMI: Body mass index. GP: general practitioner. ED: emergency department. OLS: Ordinary least squares. For details of how outcomes are calculated and ordered, see Table 1. Predictors are taken from the wave prior to outcome.

Data preparation was done in Stata (version 15) ^35^, and the simulations run in C++. For this methods illustration paper, simulations were performed 25 times in Monte Carlo simulation.

***Uncertainty and bias.*** We identified several potential sources of uncertainty with each of the data files employed. With respect to the stock population, there is uncertainty in sampling. TILDA recruited a population-representative sample of community-dwelling adults but there is inevitable variation between the sample and the population, and this variation may increase over time through attrition if those who die or drop out of the survey differ systematically from those who remain. We addressed this uncertainty, and the concomitant risk of bias, through sampling weights that used the CSO census to calculate based on age, sex, education and marital status, the probability of any given participant having been included in the sample.

With respect to transition models, the Markov regressions deliver associations between predictors and outcome that are inherently uncertain and have attendant confidence intervals. In this iteration of the model we used random draw. When the transition models were combined with the stock population to estimate biennial outcomes, changes in state were estimated by random sampling from a probability distribution, where this distribution represents the probability of the outcome in two years’ time, given their characteristics today. We managed this uncertainty by rerunning the scenarios multiple times and reporting the range of output values as confidence intervals.

With respect to replenishment, we used CSO Census data to ensure that the numbers of 50-51-year-olds added to the simulation reflected the proportion of the total 50+ population in this age bracket at each specific wave. In the model reported here we did not otherwise adjust the replenishing sample characteristics (e.g. to incorporate cohort effects of increased education access, declining tobacco consumption).

***Missing data.*** There are three potential sources of missingness in our data. First, death outside the scope of GRO linkage, which is complete in TILDA to March 2018. Since Wave 5 was conducted in 2018, at time of writing we had a mortality file providing near-full coverage of death dates within Ireland during the study period (via GRO) and additional non-comprehensive information on deaths outside the State (from family members). It is possible that we were missing a small number of deaths that occurred in Ireland after March 2018 but before the end of Wave 5 data collection in 2018, and that we were missing an unknown number of deaths that occurred outside Ireland and had not been identified via interactions with family members. In either case, this outcome was not accounted for in the current model. 

Second, an individual did not participate in one or more waves from Wave 2 onwards, either because they declined to participate or could not be contacted. If the participant left the study and did not return, then for all subsequent waves age, sex, education and mortality were treated as known but all other factors in Table 1 and Table 2 were unobservable in all waves from that point. If the participant left the study (e.g. missed Wave 2) and returned (e.g. participated in Wave 3) then age and negative diagnoses at later waves (“Have you ever had a diagnosis of cancer?” – “No”) were backfilled to missed prior waves and all other factors in Table 1 and Table 2 were unobservable in those waves that were missed. Third, an individual participated in a wave but refused to answer a question or replied “Don’t know”. In primary analyses, all such answers were treated as missing.

We quantified missingness in predictors and outcomes in reporting our results. We checked the robustness of our primary results to missing data through sensitivity analyses where the predictors were restricted to age, sex and education, where sex and education at baseline were treated as fixed, and age among non-participants in a given wave was calculated using their age at baseline and mean interview date among participants in that wave. 

Finally, we note one final data limitation in this paper: the transitions models were run on the full TILDA dataset (Waves 1–5) and use all available variables; the stock population was drawn from the TILDA public dataset, which censors some variables that we use, including age over 80 and total number of ADLs. Any predictors in the models must therefore be available in the public dataset. This limitation arises from temporary technical delays running the full simulation internally at TILDA and will be eliminated in future iterations.

## Results

### Baseline data

The key characteristics of the baseline TILDA sample are summarised in Table 3. The majority (57%) were aged under 65, a further quarter (27%) were aged 65 to 74, and 17% were aged 75+. The sample was 54% female and 46% male, reflecting sex distribution in the population and life expectancy differentials by sex.

**Table 3.** Key characteristics at Wave 1 baseline (n=8,174).

Variable		Female	Male	All
**Sex**		4,430 (54)	3,744 (46)	**8,174**
**Age (Yrs)**	*50-54*	895 (20)	726 (19)	**1,621 (20)**
	*55-59*	916 (21)	735 (20)	**1,651 (20)**
	*60-64*	778 (18)	616 (16)	**1,394 (17)**
	*65-69*	608 (14)	591 (16)	**1,199 (15)**
	*70-74*	488 (11)	477 (13)	**965 (12)**
	*75-79*	386 (9)	329 (9)	**715 (9)**
	*80-84*	222 (5)	174 (5)	**396(5)**
	*85+*	137 (3)	94 (3)	**231 (3)**
	*Missing*	*0 (0)*	*1 (<1)*	***1 (<1)***
**Education**	*Primary/none*	1,256 (28)	1,248 (33)	**2,504 (31)**
	*Secondary*	2,616 (59)	1,896 (51)	**4,512 (55)**
	*Tertiary/higher*	555 (13)	598 (16)	**1,153 (14)**
	*Missing*	*3 (<1)*	*1 (<1)*	***4 (<1)***
**BMI**	*Mean (St Dev)*	26.4 (5.6)	28.2 (3.9)	**27.2 (4.9)**
	*Missing*	*1,254 (28)*	*1,048 (28)*	***2,302 (28)***
**Smoking**	*Current*	810 (18)	680 (18)	**1,490 (18)**
	*Past*	1,387 (31)	1,730 (46)	**3,117 (38)**
	*Never*	2,233 (50)	1,333 (36)	**3,566 (44)**
	*Missing*	*0 (0)*	*1 (<1)*	***1 (<1)***
**Diagnoses**	*Diabetes*	266 (6)	368 (10)	**634 (8)**
	*Missing*	*5 (<1)*	*7 (<1)*	***12 (<1)***
	*Cancer*	306 (7)	206 (6)	**512 (6)**
	*Missing*	*1 (<1)*	*3 (<1)*	***4 (<1)***
	*Lung disease*	186 (4)	144 (4)	**330 (4)**
	*Missing*	*1 (<1)*	*3 (<1)*	***4 (<1)***
	*Hypertension*	1,651 (37)	1,380 (37)	**3,031 (37)**
	*Missing*	*5 (<1)*	*7 (<1)*	***12 (<1)***
	*Heart disease*	721 (16)	836 (22)	**1,557 (19)**
	*Missing*	*5 (<1)*	*7 (<1)*	***12 (<1)***
	*Stroke*	60 (1)	73 (2)	**133 (2)**
	*Missing*	*5 (<1)*	*7 (<1)*	***12 (<1)***
ADLs (/3) *	*No ADLs*	3,716 (94)	3,164 (95)	6,880 (95) *
	*1 ADL*	140 (4)	95 (3)	**235 (3)**
	*2 ADLs*	44 (1)	38 (1)	**82 (1)**
	*3 ADLs*	42 (1)	19 (1)	**61 (1)**
	*Missing*	*31 (<1)*	*30 (<1)*	***61 (2)***
**IADLs (/5)**	*No IADLs*	4,148 (94)	3,568 (95)	**8,170 (94)**
	*1 IADL*	166 (4)	101 (3)	**267 (3)**
	*2+ IADLs*	115 (3)	72 (2)	**187 (2)**
	*Missing*	*1 (<1)*	*3 (<1)*	***4 (<1)***

**Legend:** For categorical variables, cells are number of people (%). For continuous and count variables, cells are mean (standard deviation). BMI: Body mass index. (I)ADLs: (Instrumental) Activities of Daily Living. For definitions and sources, see Table 2. * ADL count is taken from Wave 2 due to an inconsistency in how questions are asked between Wave 1 and subsequent Waves.

Fewer than a fifth (18%) of participants were active smokers at enrolment but a further 38% had smoked in the past. Of the six chronic diseases that we modelled, the most prevalent were hypertension (37%) and heart disease (19%). All other prevalences were between 2% (stroke) and 8% (diabetes). For both ADLs and IADLs, 95% of the sample reported no problems at baseline.

### Longitudinal and outcome data

***Mortality and attrition.*** Sample sizes, mortality and attrition are presented in Table 4. Of the 8,174 participants at Wave 1, 208 (2.5%) died by Wave 2 and 972 (11.9%) did not participate in Wave 2. An additional 288 participants were added to the sample at Wave 2. Between 261 and 320 participants died by wave in Waves 3–5, and an additional 187 participants joined the sample. Of 8,649 participants in at least one wave aged 50+, 2,364 (27%) did not participate in Wave 5 but were not identified as deceased and are therefore assumed to be alive.

**Table 4.** Outcomes: participation, attrition and mortality, Waves 1-5.

	Wave 1	Wave 2	Wave 3	Wave 4	Wave 5
Participated	8,174	7,282	6,619	5,942	5,213
No participation, assumed alive		972	1,440	1,904	2,364
Deceased (cumulative)		208	528	789	1,072
**TOTAL**	**8,174**	**8,462**	**8,587**	**8,635**	**8,649**

**Figure 3.**
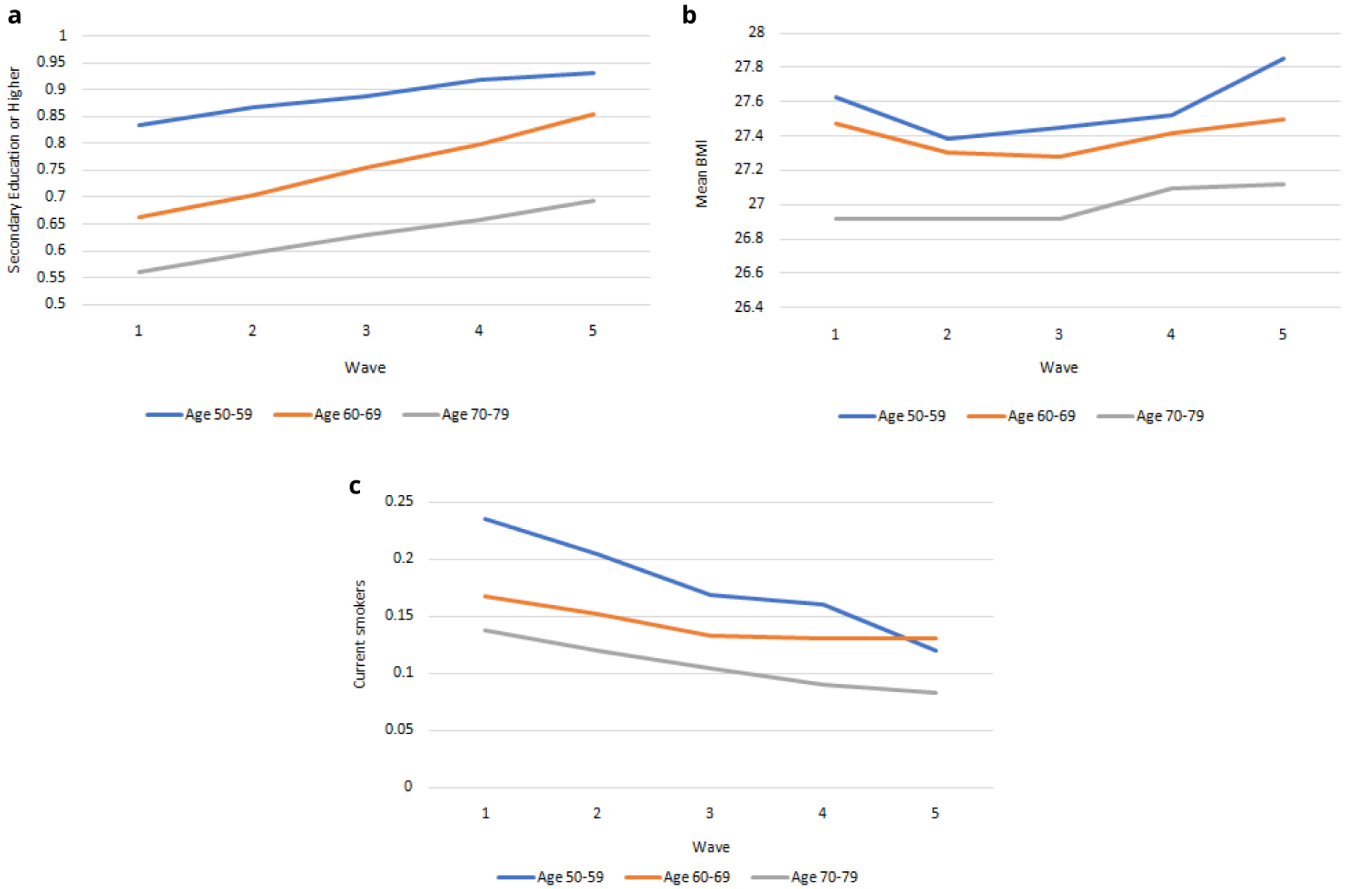
**a**. Secondary education or higher. **b**. BMI. **c**. Current Smoker. x-axis: Wave of TILDA; y-axis: Mean at specified ages. Source: TILDA full dataset.

***Risk factors.*** In Figure 3 we compare education, smoking and BMI for 50-59-year-olds, 60-69-year-olds and 70-79-year-olds from Waves 1 to 5. These variables are risk factors for health and related outcomes, and vary across age cohorts. Therefore as the population ageing depicted in Figure 2 occurs, the risk of specific diseases and limitations will change for a given age over time as the risk factors change. In Figure 3a we see that the level of education achieved was higher among younger age groups and increases for all ages from Wave 1 to Wave 5. In Figure 3b we see that BMI was lower among older age groups and mean BMI was slightly higher for each age group at Wave 5 than at Wave 1. In Figure 3c we see that the level of smoking was higher among younger age groups and falling more quickly among younger age groups from Wave 1 to Wave 5. The future health and associated outcomes of the TILDA cohort and older Irish population will reflect these trends as the older Irish population is increasingly better educated, less likely to smoke, and with higher BMI.

**Figure 4.**
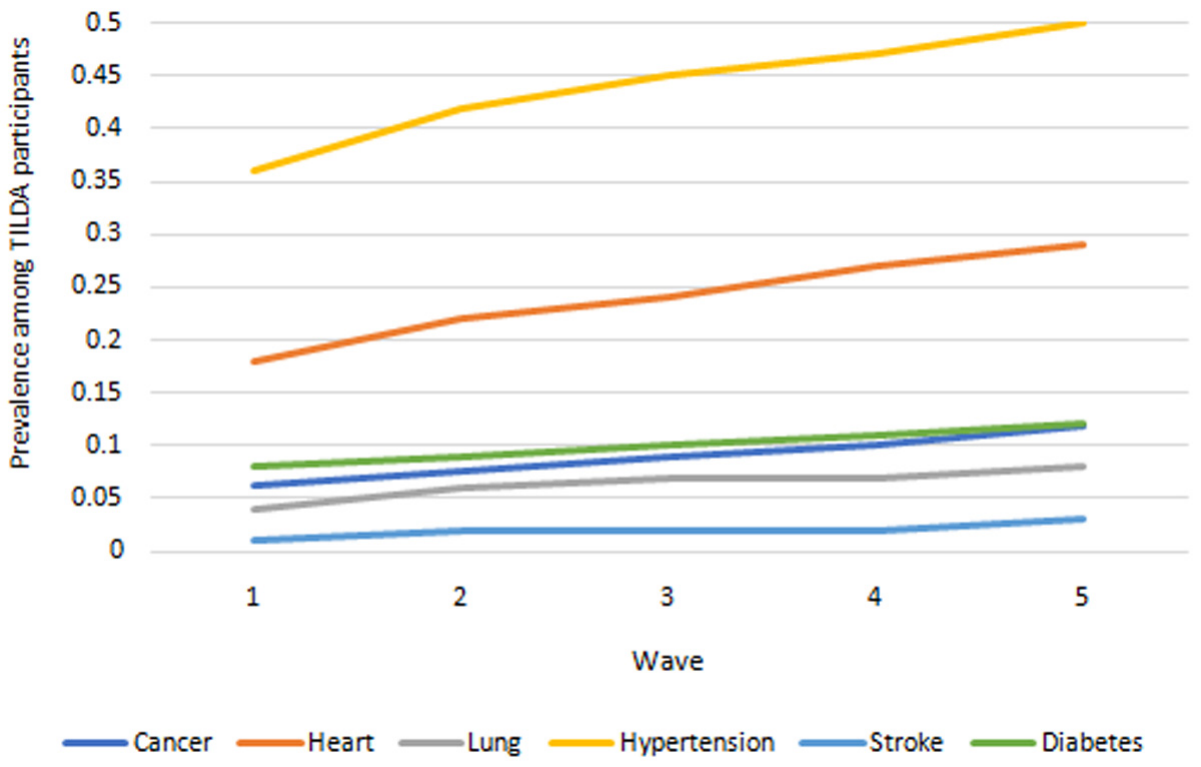
Prevalence of serious diseases. Source: TILDA full dataset.

***Diagnoses.*** Prevalences for each of six chronic diseases are presented in Figure 4. Highest prevalence across waves was consistently among hypertension and heart disease, and lowest among stroke and lung disease. Prevalence of all conditions increased over time.

**Figure 5.**
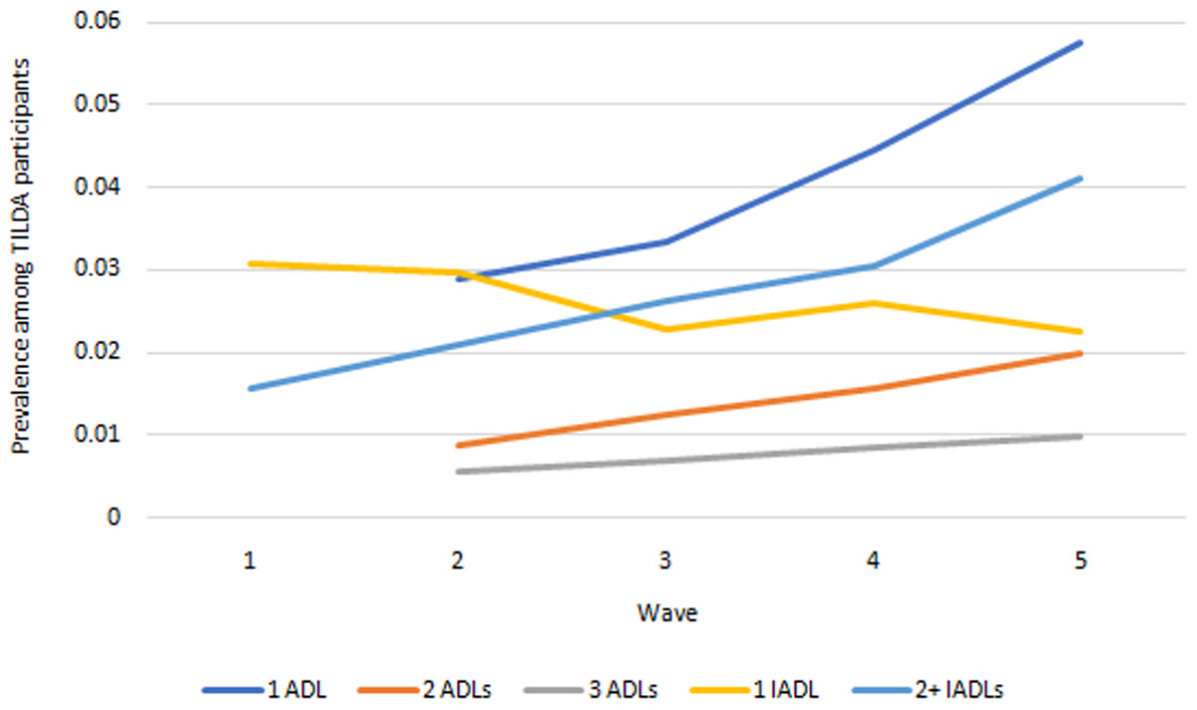
Prevalence of functional limitations. Source: TILDA full dataset. ADLs Wave 2 onwards only due to measurement inconsistency at Wave 1 (see Table 1>Notes).

***Functional limitations.*** Prevalence of ADLs and IADLs are presented in Figure 5. The proportions of people with one, two and three ADLs all increased across waves. The proportions of people with precisely one IADL fell across waves but at a slower rate than the proportion of those with two or more IADLs, indicating an increase in the total IADL burden also.

**Figure 6.**
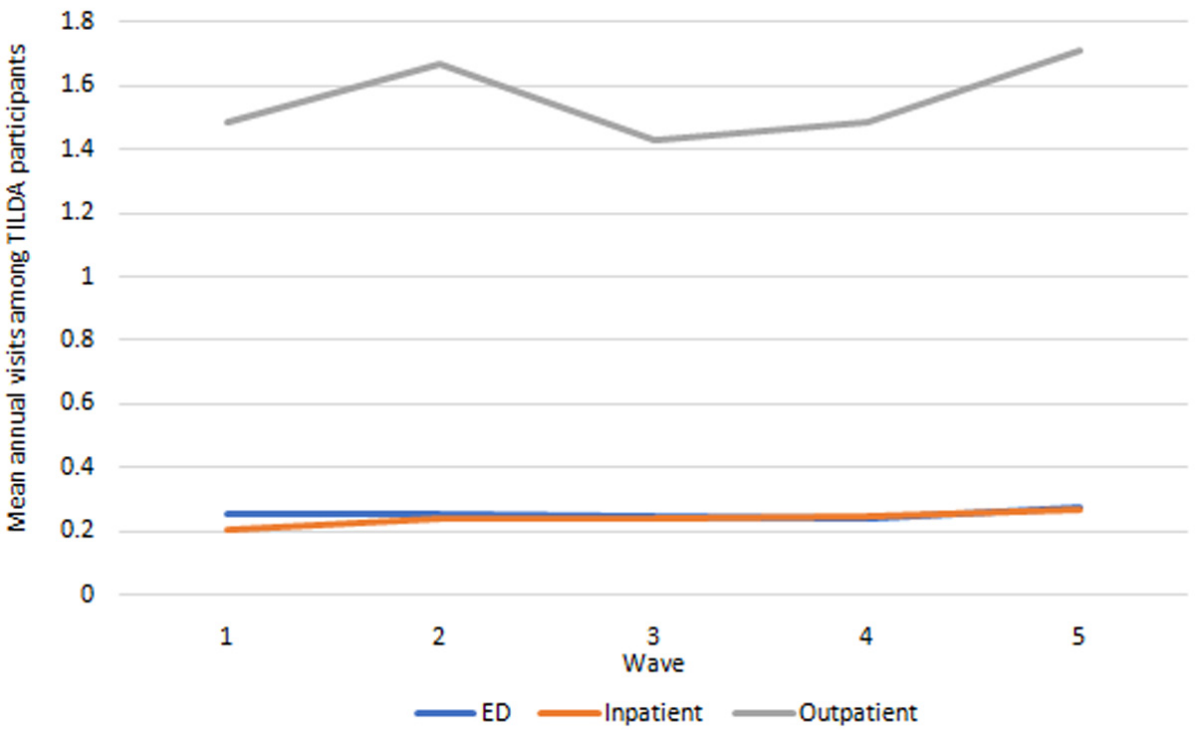
Health care utilisation. Source: TILDA full dataset.

***Health care utilisation.*** Health care use outcomes are presented in Figure 6. Mean hospital visits (ED, outpatient, inpatient) all exhibited small increases between Wave 1 and Wave 5.

***Missing outcome data.*** Missing responses on outcomes among participants by Wave are presented in Table 5. For CAPI responses on diagnoses, functional limitations and health care use at Wave 1 there was mean missingness of <0.5%. From Waves 2 to 5 there was mean missingness of 2% to 5%. One exception with respect to missingness was BMI. At Wave 1 there was BMI data only among those who participated in the nurse-led health assessment centre so there was 28% missingness. Missing BMI data remained higher Wave 2 onwards when this was added to the CAPI (6%-12%).

**Table 5.** Outcomes: missingness among participants, Waves 1-5.

	Wave 1	Wave 2	Wave 3	Wave 4	Wave 5
**Participants**	**8,174**	**7,282**	**6,619**	**5,942**	**5,213**
*Risk factors*					
BMI	28%	6%	8%	10%	12%
Stop smoking	<0.5%	2%	3%	4%	4%
					
*Diagnoses*					
Diabetes	<0.5%	2%	4%	4%	5%
Cancer	<0.5%	2%	3%	4%	4%
Lung disease	<0.5%	2%	3%	4%	4%
Hypertension	<0.5%	2%	3%	4%	4%
Heart disease	<0.5%	2%	3%	4%	4%
Stroke	<0.5%	2%	4%	4%	5%
					
*Functional limitations*					
ADLs	<0.5%	2%	3%	4%	4%
IADLs	<0.5%	2%	3%	4%	4%
					
*Health care utilisation*					
ED visits	<0.5%	2%	3%	4%	4%
Inpatient admissions	<0.5%	2%	3%	4%	4%
Outpatient visits	<0.5%	2%	3%	4%	5%

**Notes:** Mortality was treated as never missing: if GRO linkage had not identified a participant as deceased at a given Wave they were assumed alive irrespective of participation.

### Main results

**Figure 7.**
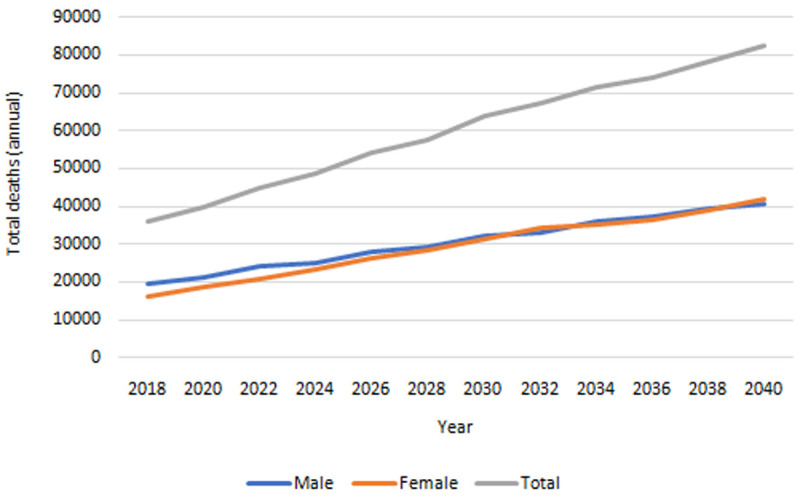
Projected total deaths among people in Ireland aged 50+. Source: Authors’ own IFOAM calculations.

***Mortality.*** Our IFOAM population-level projections for mortality 2018-2040 are provided in Figure 7. The total number of deaths is projected to increase from approximately 36,000 in 2018 to over 82,000 in 2040. The gender gap, which currently sees more deaths among males than females, is projected to dwindle over time.

**Figure 8.**
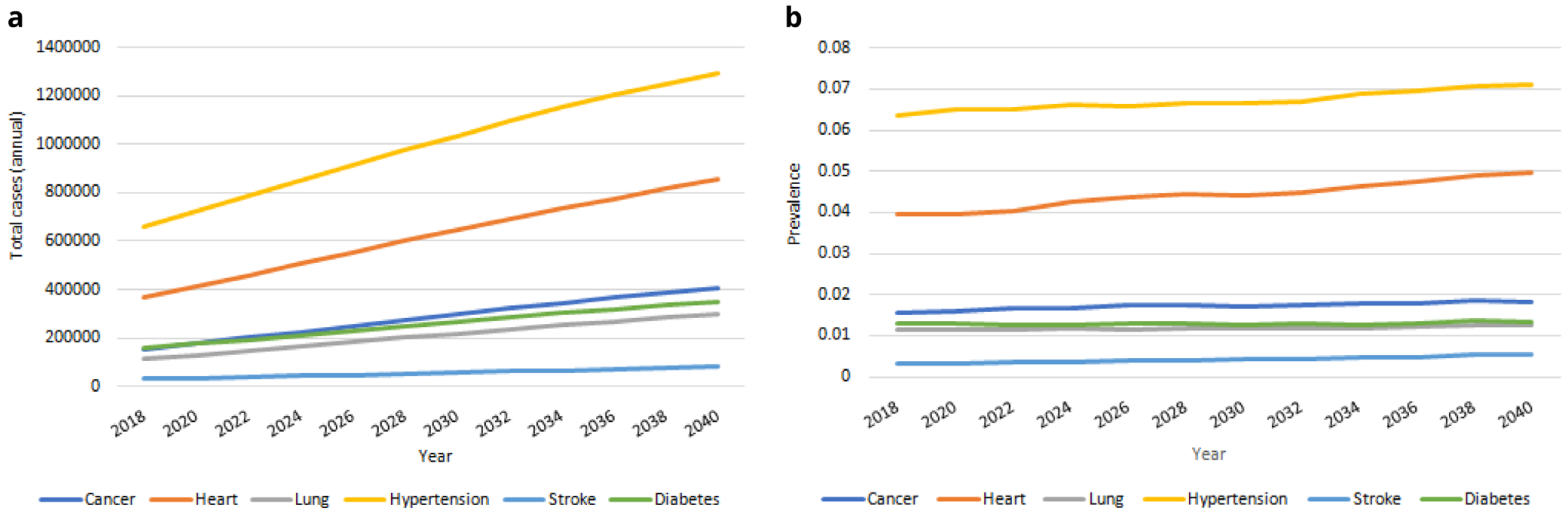
Serious diseases **a** Total cases **b** Prevalence.

***Diagnoses.*** Our IFOAM population-level projections for serious chronic diseases from 2018-2040 are provided in Figure 8a. We estimate that in 2040 there will be more than 400,000 people aged over 50 in Ireland with cancer; over 850,000 with heart disease; approximately 300,000 with lung disease; approximately 1,300,000 with hypertension; more than 80,000 with stroke and approximately 350,000 with diabetes. These represent large proportionate rises in the total number of people with each of the six conditions from 2018, from 96% (hypertension) to 171% (stroke). The corresponding rate of incidence for these diseases is provided in Figure 8b. This incidence increases for all six diseases over time, but at notably slower rates, from 4% (diabetes) to 63% (stroke). The large increases in Figure 8a are therefore predominantly accounted for by the increasing numbers of people aged 50+, and to some extent by the increasing average age and so higher risk of serious disease among those older people (these two trends were first illustrated in Figure 1).

**Figure 9.**
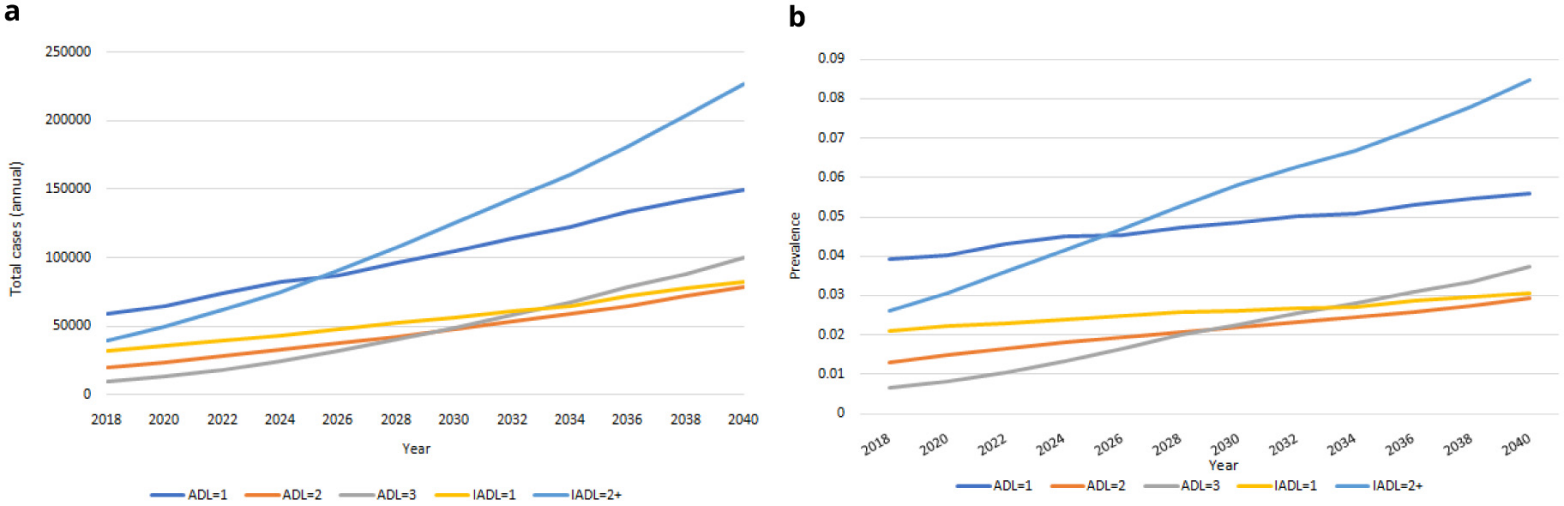
Functional limitations **a** Total cases **b** Prevalence.

***Functional limitations.*** Our IFOAM population-level projections for functional limitations from 2018-2040 are provided in Figure 9. We estimate that in 2040 there will be more than 635,000 people aged over 50 in Ireland with at least one functional limitation, up from an estimated 160,000 in 2018. These represent large proportionate rises in the total number of people for each group. The corresponding rates of prevalence are provided in Figure 9b. Prevalences will increase for all groups over time, but at slower rates. Increases in Figure 9a are therefore predominantly accounted for by the increasing numbers of people aged 50+, and to some extent by the increasing average age and so higher risk of functional limitations among those older people (these two trends were first illustrated in Figure 1).

**Figure 10.**
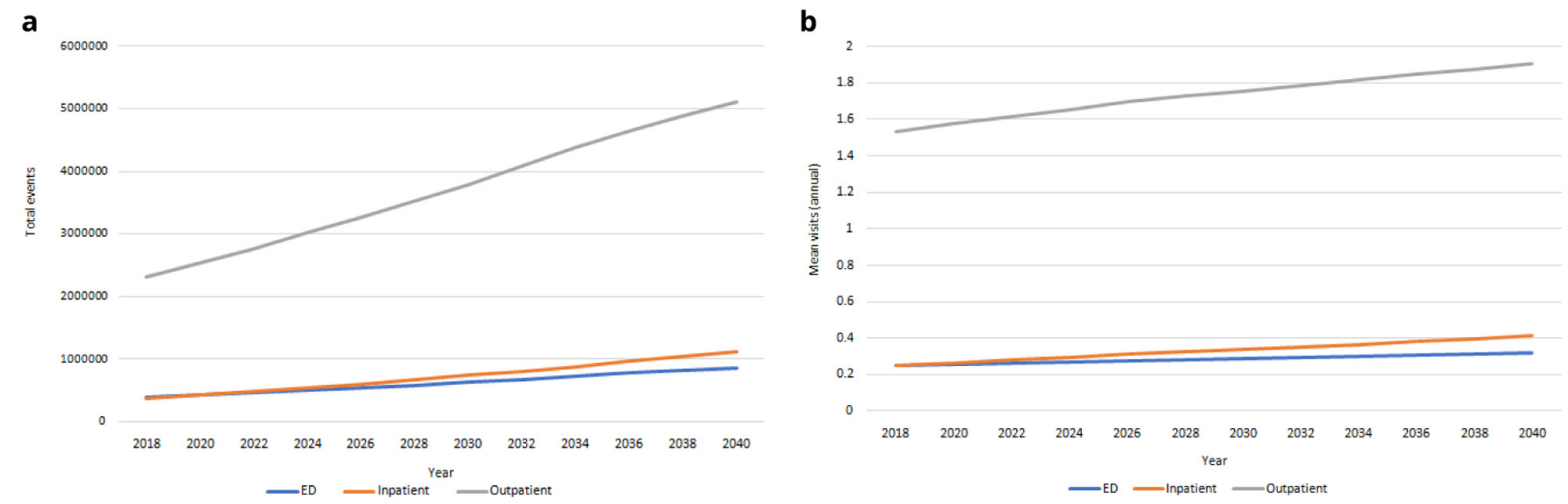
Health care utilisation **a** Total admissions **b** Mean per capita use.

***Health care utilisation.*** Our IFOAM population-level projections for hospital use from 2018-2040 are provided in Figure 10. We estimated there will be more than 850,000 ED admissions for people aged 50+ in 2040; more than 1,100,000 inpatient stays and more than 5,100,000 outpatient appointments. These represent increases of 121%-200% on 2018 levels. This incidence will increase for all groups over time, but at notably slower rates. Increases in Figure 10a are therefore predominantly accounted for by the increasing numbers of people aged 50+, and to some extent by the increasing average age and so higher risk of hospital visits among those older people (these two trends were first illustrated in Figure 1).

**Figure 11.**
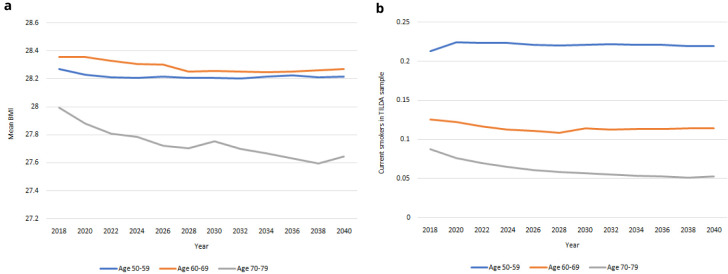
Risky behaviours **a** BMI **b** Prevalence of smoking.

***Risk factors.*** Population-level projections for risk factors from 2018-2040 are provided in Figure 11. In Figure 11a we project a steady decrease in mean BMI in the older population, occurring at a similar rate of decrease for males and females, with mean BMI remaining more or less constant in the younger age bands. In Figure 11b we project decreases in smoking prevalence in the oldest age group and constant smoking prevalence in the youngest age group.

### Supplementary materials

In the online supplementary materials we provide the regression output for all transition models (per Table 2), as well as summary information on three types of validation and checking that we performed on our results.

**Figure 12.**
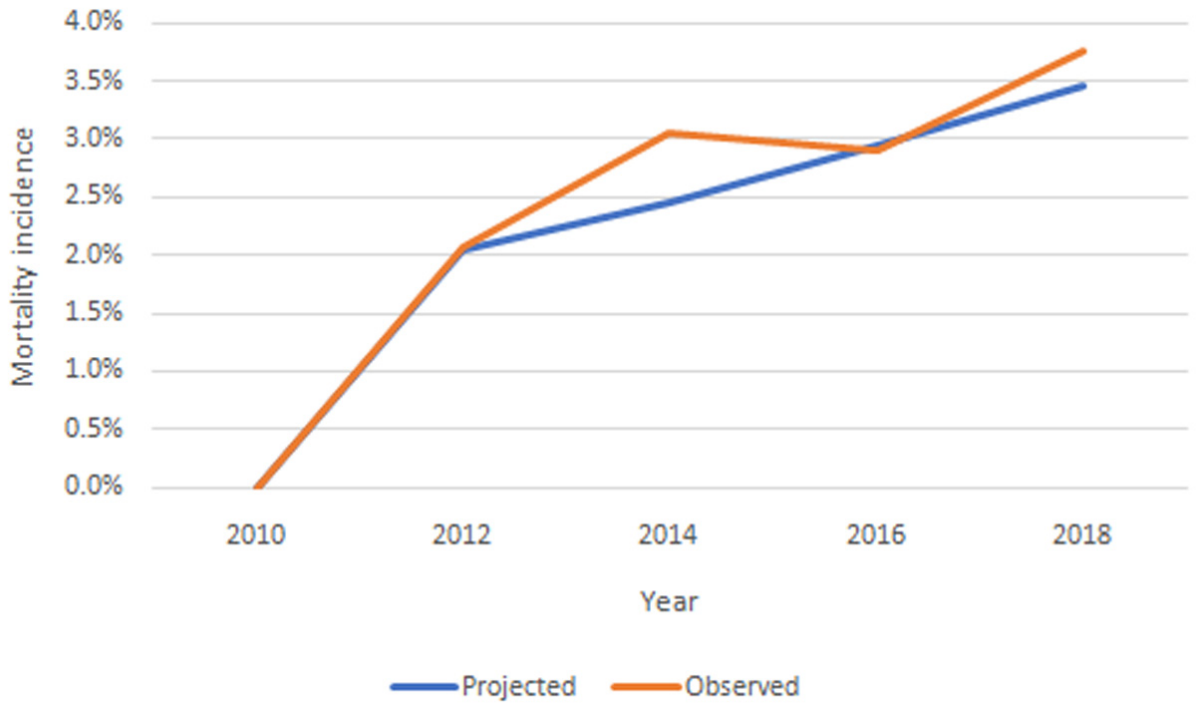
Observed and projected incidence of mortality wave by wave. Source: TILDA and authors’ own IFOAM calculations.

***Internal validation.*** For internal validation we compared the observed values in the TILDA dataset with our IFOAM projections from Wave 1 (2009-2011) to Wave 5 (2018). For example, in Figure 12 we present the observed and projected incidence of mortality wave by wave. We consider that the model has good predictive accuracy against observed values.

Equivalent figures for all diagnoses, functional limitations, risk factors and health care use are provided in the Appendix. In all cases we consider the model has good predictive accuracy against observed values.

***External validation.*** For external validation we would like to compare our IFOAM projections from Wave 1 (2009-2011) to Wave 5 (2018) versus observed values for the same variables in the same population in the same time period measured by some third party. We have not been able to identify any publicly available data that meet these criteria, but some ongoing considerations are summarised in the Appendix.

***External corroboration.*** We sought data sources that for 2020 onwards projected the same variables in the same population as TILDA. We term this *corroboration*, not *validation*, because there is no way to know how accurate are the IFOAM projections or any other set of projections when compared against each other. We identified projections for mortality from the CSO and no other projections that were directly comparable to those reported in this paper, and details are provided in the Appendix.

## Discussion

### Key findings

We have developed and validated a microsimulation model that predicts future health and related outcomes among older people in Ireland. The results suggest that in the next 20 years there will be large increases in Ireland in the number of people dying, in the number of people living and dying with serious illness and functional impairment, and in demand for hospital care. The most important driver of these increases is the absolute number of older people in Ireland as the population, which is young compared to other high-income countries, ages. Additionally, the increasing proportion of older old and oldest old citizens will necessarily increase the average prevalence of certain conditions and limitations.

These results are broadly consistent with prior projections on mortality, serious chronic disease and health care use ^11, 24, 36^. The IFOAM model complements and extends prior work in a number of important ways. First, TILDA data provide a range of outcome variables that are not otherwise collected routinely or repeatedly in Ireland. This equips us to quantify today and predict the future prevalence of these outcomes for the first time. Second, by using microsimulation we begin to delineate the interaction of individual-level health and related outcomes. This strengthens our prediction capacity, both because these relationships are complex and because they are changing over time (50-year-olds in 2030 will not be identical to 50-year-olds in Wave 1 in 2010). Third, since TILDA is part of an international series of studies and the IFOAM now joins an international family of microsimulation models, we are able to contextualise the Irish situation against international comparators and work across countries to tackle the global challenges of population ageing.

### Limitations

In this paper we report a version of the IFOAM that uses the public TILDA dataset for its stock population. The public TILDA dataset censors some data and our choice of predictors is restricted as a consequence. We will address this in the next iteration of the model, which will run on the full TILDA dataset. The TILDA population was representative of the older community-dwelling population at Wave 1 in 2010 and this constrains our ability to model those outcomes that are strongly associated with residential care living: old age, greater frailty and multimorbidity. These constraints diminish as the TILDA sample ages and we will monitor future projects to understand results in the context of potentially undercounting older residential care. In the model reported here we did not otherwise adjust the replenishing sample characteristics (e.g. to incorporate cohort effects of increased education access, declining tobacco consumption). We will explore approaches to this in future iterations.

The lack of suitable comparable data sources limits our capacity to do external validation (to 2020) and corroboration (beyond 2020) ^37, 38^. For more details, see Appendix in the Supplementary Materials. TILDA data are all self-reported, which may result in some measurement error or biases. 

### Planned future iterations

The aim of this paper was to document our IFOAM methods and the face validity of the projections that we generate. There are two ongoing projects that will apply the IFOAM. First, in collaboration with the Irish Department of Health, we will project future population health need and health care use among older people with palliative care needs in Ireland (project ID: HRB/ARPP/2018/A/005). Second, in collaboration with the University of Southern California, we will examine methods and results in projecting future prevalence of dementia in Ireland and the US (NIA/P30AG066589).

Prior to finalising those analyses we will make various updates to the IFOAM methods. We will run the projections on the full TILDA dataset, to circumvent the limitations of the public dataset, and to incorporate Wave 6 data, which will soon become available. TILDA-GRO linkage is scheduled for an update in 2022 and in future iterations we will update the model accordingly. TILDA collects usage across more than 20 categories of health care use ^39^. We will incorporate all categories in future models, and then combine with unit costs for each category to model health care costs ^40^. In this iteration of the model we used random draw to manage uncertainty in modelling the transitions; in future iterations we will incorporate nonparametric bootstrapping.

Finally, we will identify opportunities to apply the model beyond currently funded grants. The US FEM additionally models household economic outcomes: income, pension and assets. These are beyond the scope of our current IFOAM project but we have the possibility to extend to these outcomes in future.

## Conclusion

We have developed and validated a microsimulation model that predicts future health and related outcomes among older people in Ireland. The results highlight profound challenges for Irish health care and wider society. This paper provides a detailed overview of the rationale and methods for this work. Future papers will address identified policy questions in Ireland and internationally. The model enhances the capacity of researchers and policymakers to quantify future health and economic dynamics among older people in Ireland, and to evaluate ex ante policy responses to these dynamics.

## Data availability

Researchers interested in using regular waves of TILDA data may access the data for free from the following sites: Irish Social Science Data Archive (ISSDA) at University College Dublin ( http://www.ucd.ie/issda/data/tilda/); Interuniversity Consortium for Political and Social Research (ICPSR) at the University of Michigan ( http://www.icpsr.umich.edu/icpsrweb/NACDA/studies/34315). 

Replication of the results reported in this article requires access to the full TILDA dataset, which is held on secure servers at the study site at Trinity College Dublin (TCD). Researchers seeking access to the full TILDA dataset may apply to access the data on the TCD campus (tilda.tcd.ie); applications are considered on a case-by-case basis; all Stata do files and code employed in this paper will be made available to applicants on request.

### Reporting guidelines

Open Science Framework: Appendix to: [Projecting future health and service use among older people in Ireland: an overview of a dynamic microsimulation model in The Irish Longitudinal Study on Ageing (TILDA)], https://doi.org/10.17605/OSF.IO/GT2SH.

Data are available under the terms of the Creative Commons Zero "No rights reserved" data waiver (CC0 1.0 Public domain dedication).

## Consent

Ethical approval for each wave of the TILDA study is obtained from the Faculty of Health Sciences Research Ethics Committee in Trinity College Dublin. Participants are provided with sufficient information to make an informed decision about their participation including advance notice of the study. Written consent is obtained for separate components of the study (i.e. interview, health assessment, blood samples); participants may refuse to take part in or withdraw at any time without justification. Ethical approval for the secondary analysis of TILDA data used in this study was part of this overall approval.
