# Supplementary material for: Projecting future health and service use among older people in Ireland: an overview of a dynamic microsimulation model in The Irish Longitudinal Study on Ageing (TILDA)
Source: HRB Open Res. 2022 Jun 10;5:21. Originally published 2022 Mar 21. [Version 2] doi: 10.12688/hrbopenres.13525.2 (PMC9554695; doi:10.12688/hrbopenres.13525.2)
Supplement: Peer review history

# Peer review: Reviewer response for version 2

**DOI:** 10.21956/hrbopenres.14828.r32288

**Published:** 2022-06-20

**Authors:** Deborah Schofield

**Affiliations:** 1 Centre for Economic Impacts of Genomic Medicine, Macquarie University, Sydney, NSW, Australia

I am satisfied with the authors' response and for the paper to proceed to indexing.

Is the rationale for developing the new method (or application) clearly explained?

Yes

Is the description of the method technically sound?

Yes

Are the conclusions about the method and its performance adequately supported by the findings presented in the article?

Yes

If any results are presented, are all the source data underlying the results available to ensure full reproducibility?

Partly

Are sufficient details provided to allow replication of the method development and its use by others?

Yes

Reviewer Expertise:

Microsimulation and health/ageing/social services

I confirm that I have read this submission and believe that I have an appropriate level of expertise to confirm that it is of an acceptable scientific standard.

# Peer review: Reviewer response for version 1

**DOI:** 10.21956/hrbopenres.14761.r31701

**Published:** 2022-04-27

**Authors:** Conor Keegan

**Affiliations:** 1 Social Research Division, Economic and Social Research Institute, Dublin, Ireland; 2 Trinity College Dublin, Dublin, Ireland

This paper describes the development and validation of a demographic-economic microsimulation model for the Irish population aged 50 and over. The model is primarily populated using The Irish Longitudinal Study on Ageing (TILDA) dataset. The model projected large increases in total deaths, serious illness and functional impairment, and demand for hospital care between 2018 and 2040. The authors consider the model to perform well in terms of internal validation but the lack of suitable comparable data sources limited the ability to externally validate. Although restricted to modelling dynamics for those aged 50 and over only, which may limit its applications, given the challenges posed by demographic ageing the model should have practical policy-relevant uses. The paper is well-written and well-structured.

 Page 3, Methods section, paragraph 1: ‘A younger population translates to fast-growing population health need:’ – is this saying that younger populations may age more quickly and therefore growth in need is greater than for elderly populations? If so, is there evidence behind this? Perhaps make this statement a little clearer.

 Page 5, Table 1: Is there a reason additional measures of healthcare utilisation are not captured? From my understanding, TILDA captures a wide variety of metrics on community and social care utilisation.

 Page 7, paragraph 1: It seems to me that an additional source of bias may relate to the fact that a number of variables used in the model are self-reported. How big a concern is this for the modelling? For instance, is recall bias an issue regarding the measure of healthcare utilisation? And might this differ systematically across groups in the sample (e.g. older groups, heavy user groups)?

 General method comment: While increases in the number of older individuals may be driving increases in service demand, this is probably quite a pessimistic view of ageing – a 65-year-old in 20 years’ time will likely have a very different utilisation profile compared with a 65-year-old today. Does the model incorporate or allow for changes in age-specific utilisation in the future? For example, is there an interaction within the model that refines the ageing effect by combining with changes in age-specific trends in disease prevalence/risk factors, etc.?

 General method comment: In the model, you describe managing statistical uncertainty due to sampling variation, but inherent in any projections are also uncertainties around future trends in key drivers (e.g. ageing, disease prevalence etc.). It might be useful rather than projection on point estimates (e.g. 850,000 ED admission by 2040) that you project on a range capturing varying assumptions in relation to key drivers. This would better acknowledge uncertainty in relation to future trends and would provide more informative output for policymakers.

 Page 12, final paragraph: In terms of describing the healthcare utilisation projections – I’m not sure it’s accurate to say there will be X number of OPD appointments etc. in 2040. Realised utlisation of services in the future will be a function of capacity in the system to deliver care. What’s been captured is projected demand for services based on underlying drivers (e.g. ageing). However, this is still incomplete as there is no allowance made for unmet demand for services. From a policy perspective, what matters is the total requirements for care – is there any way that the model can be refined to include unmet demand as part of the projections?

 Page 14, Discussion section: The authors describe the absolute increase in the older population as the most important driver of these increases. While I don’t doubt this – there is no evidence provided to back up this assertion. Could the authors comment on the other drivers and their relative impact? Is there any way within the model to decompose the contribution of different drivers to projected changes in the respective outcomes?

 Page 14: In terms of external validation of healthcare utilisation particularly, the authors should bear in mind that comparing projected demand from recent years with actual realised utilisation may not be that informative. Actual utilisation, given capacity constraints, is likely not an accurate reflection of increased demand and it might be worthwhile also capturing changing unmet demand (waiting list) data in comparisons. 

 Minor comment: In places (including the title), you use the term ‘predicts future’ or ‘projects future’ – the word future is probably not needed. There is also a difference between a projection and a prediction/forecast.

Is the rationale for developing the new method (or application) clearly explained?

Yes

Is the description of the method technically sound?

Yes

Are the conclusions about the method and its performance adequately supported by the findings presented in the article?

Yes

If any results are presented, are all the source data underlying the results available to ensure full reproducibility?

No

Are sufficient details provided to allow replication of the method development and its use by others?

Partly

Reviewer Expertise:

Healthcare projection modelling, healthcare utilisation, health care policy, health economics

I confirm that I have read this submission and believe that I have an appropriate level of expertise to confirm that it is of an acceptable scientific standard.

# Response

**Published:** 2022-05-30

**Authors:** Peter May

**Affiliations:** Trinity College Dublin, Ireland

Thanks to the reviewer for their detailed comments. We have numbered (R2.1, R2.2, etc) and responded to specific comments below marked *>, and we have revised the manuscript accordingly. The paper is now stronger as a result. Cross-references to R1.1, R.1.2, etc are included where the comment corresponds to something similar said by Reviewer 1. 

 R2.1 Page 3, Methods section, paragraph 1: ‘A younger population translates to fast-growing population health need:’ – is this saying that younger populations may age more quickly and therefore growth in need is greater than for elderly populations? If so, is there evidence behind this? Perhaps make this statement a little clearer. 

 *>Clarification added: 

 the proportional increase in need for services used by older people will be larger in countries that have yet to experience population ageing compared to those countries where the current population is already on average older. 

 R2.2 Page 5, Table 1: Is there a reason additional measures of healthcare utilisation are not captured? From my understanding, TILDA captures a wide variety of metrics on community and social care utilisation. 

 *>Per response to Reviewer1/Comment15 and Discussion>Future iterations (“TILDA collects usage across more than 20 categories of health care use…”) TILDA collects a huge amount of data on health and health care use. Modelling all possible outcomes is not feasible. Our aim in this paper is to document the methods and then future papers will model specific outcomes addressing specific research questions, where these documented methods are a crucial reference point. 

 R2.3 Page 7, paragraph 1: It seems to me that an additional source of bias may relate to the fact that a number of variables used in the model are self-reported. How big a concern is this for the modelling? For instance, is recall bias an issue regarding the measure of healthcare utilisation? And might this differ systematically across groups in the sample (e.g. older groups, heavy user groups)? 

 *>We acknowledge this in the Limitations: “TILDA data are all self-reported, which may result in some measurement error or biases.” External validation of the TILDA data are complicated by the problems we note elsewhere in the paper. We have now added a sentence and reference: 

 “Previous research has quantified some potential issues in the form of ‘implausible transitions’, where self-reported responses across waves apparently contradict each other.” 

 R2.4 General method comment: While increases in the number of older individuals may be driving increases in service demand, this is probably quite a pessimistic view of ageing – a 65-year-old in 20 years’ time will likely have a very different utilisation profile compared with a 65-year-old today. Does the model incorporate or allow for changes in age-specific utilisation in the future? For example, is there an interaction within the model that refines the ageing effect by combining with changes in age-specific trends in disease prevalence/risk factors, etc.? 

 *>Clarification added in Discussion>Limitations 

 “In the model reported here we did not otherwise adjust the replenishing sample characteristics (e.g. to incorporate cohort effects of increased education access, declining tobacco consumption). We will address this in future iterations using data from Healthy Ireland and the Census to model cohort effects in our predictors.” 

 R2.5 General method comment: In the model, you describe managing statistical uncertainty due to sampling variation, but inherent in any projections are also uncertainties around future trends in key drivers (e.g. ageing, disease prevalence etc.). It might be useful rather than projection on point estimates (e.g. 850,000 ED admission by 2040) that you project on a range capturing varying assumptions in relation to key drivers. This would better acknowledge uncertainty in relation to future trends and would provide more informative output for policymakers. 

 *>See R1.8. 

 R2.6 Page 12, final paragraph: In terms of describing the healthcare utilisation projections – I’m not sure it’s accurate to say there will be X number of OPD appointments etc. in 2040. Realised utlisation of services in the future will be a function of capacity in the system to deliver care. What’s been captured is projected demand for services based on underlying drivers (e.g. ageing). However, this is still incomplete as there is no allowance made for unmet demand for services. From a policy perspective, what matters is the total requirements for care – is there any way that the model can be refined to include unmet demand as part of the projections? 

 *>Added clarification to Discussion>Limitations: 

 “Our health care utilisation projections are based on current TILDA data of realised demand for health care among older people in Ireland. That is, we model the health care that people have used; we have no measure of demand that incorporates unmet need, and we have no data on supply-side issues including workforce, skill mix, building capacity or financing.  Our hospital projections therefore entail important implicit assumptions: current capacity will expand to meet the larger number of hospital visits, but patterns of use including systemic factors such as waiting lists and entitlements are otherwise unchanged.  These assumptions may not be realistic, particularly in the context of large-scale reforms to the Irish health care system.  Applications of this model to address policy questions will have to consider the specific dynamics of supply-sides issues in relation to that question, and model different clearly defined scenarios, including the role of unmet need, accordingly.” 

 R2.7 Page 14, Discussion section: The authors describe the absolute increase in the older population as the most important driver of these increases. While I don’t doubt this – there is no evidence provided to back up this assertion. Could the authors comment on the other drivers and their relative impact? Is there any way within the model to decompose the contribution of different drivers to projected changes in the respective outcomes? 

 *>We introduce this idea in Methods>Setting and context: “There are therefore two important dynamics at play in Ireland’s population-level ageing: the total number of older people is growing rapidly; and their age distribution is shifting upwards, meaning that on average, the larger older populations aged 50+ in the next two decades will probably be older than today and – all else held constant – have higher prevalence of serious disease, functional limitations and other population health risks.” 

 This is then illustrated in the results, e.g. Figure 8 shows the total number of cases and the prevalence of specific serious illnesses. Total number of cases = prevalence * number of people at each age in the population. Rates of increase in 8a far exceed those in 8b (and similarly for other figures in the Main Results that compare rates and total cases in different outcomes). Therefore, per Main Results>Diagnoses: 

 “The large increases in Figure 8a are therefore predominantly accounted for by the increasing numbers of people aged 50+, and to some extent by the increasing average age and so higher risk of serious disease among those older people.” 

 R2.8 Page 14: In terms of external validation of healthcare utilisation particularly, the authors should bear in mind that comparing projected demand from recent years with actual realised utilisation may not be that informative. Actual utilisation, given capacity constraints, is likely not an accurate reflection of increased demand and it might be worthwhile also capturing changing unmet demand (waiting list) data in comparisons.  

 *>See R2.6. 

 R2.9 Minor comment: In places (including the title), you use the term ‘predicts future’ or ‘projects future’ – the word future is probably not needed. There is also a difference between a projection and a prediction/forecast. 

 *>We have changed predict to project in multiple places through the document, and removed the word future at some places through the document.

# Peer review: Reviewer response for version 1

**DOI:** 10.21956/hrbopenres.14761.r31698

**Published:** 2022-04-21

**Authors:** Deborah Schofield

**Affiliations:** 1 Centre for Economic Impacts of Genomic Medicine, Macquarie University, Sydney, NSW, Australia

This paper describes the development of a microsimulation model of the ageing of the Irish population. The paper is clearly written.

 More detailed comments:

Methods

1. It is unclear whether care homes are included in the model.
2. What proportion of the population has private health insurance?
3. Are carers included in the data?
4. What is the retention rate for the survey?
5. Was the mortality data linked to the survey underpinning the model - if not, what was it used for?
6. Did the data include only the six health conditions/risk factors referred to in Table 1 or was this a subset used for the model?
7. Table 2: BMI is also a risk factor for cancer and stroke; smoking is also a risk factor for heart disease and stroke.
8. How many times were the scenarios run to establish confidence intervals?
9. Are there plans to include important trends such as increasing education and declining smoking in the model?

Results

1. Diagnoses: Hypertension is typically considered a risk factor rather than a disease.
2. Missing data: The 28% missing BMI data in wave 1 is high - could this be imputed from subsequent waves assuming BMI remained similar over time? This seems important given the importance of BMI as a risk factor.
3. Missing outcome data: The last sentence "Missing BMI data remained higher Wave 2...." doesn't seem to make sense - is there a word or two missing?
4. No significance testing was reported - could this be added to comment on the importance of change over time or will this be in a future more detailed analysis paper?

Discussion

1. What will the model be used for - projecting expenditure, resource planning, etc.?
2. The model seems as though it would be useful for projecting the increasing rate of comorbidity and frailty as the population ages - important in relation to duration and cost of hospital admissions for example.
3. If there is a more comprehensive data set with, for example, 20 categories of healthcare use and perhaps many more health conditions why was it not used at the outset?
4. The paper has several particular strengths which I thought were covered quite well in the paper immediately before the **Limitations** section in the discussion, although not under the specific heading of strengths. Perhaps prefacing the following material with the heading **Strengths** and creating a new paragraph as follows would suffice:
**Strengths**
  “First, TILDA data provide a range of outcome variables that are not otherwise collected routinely or repeatedly in Ireland. This equips us to quantify today and predict the future prevalence of these outcomes for the first time. Second, by using microsimulation we begin to delineate the interaction of individual-level health and related outcomes. This strengthens our prediction capacity, both because these relationships are complex and because they are changing over time (50-year-olds in 2030 will not be identical to 50-year-olds in Wave 1 in 2010). Third, since TILDA is part of an international series of studies and the IFOAM now joins an international family of microsimulation models, we are able to contextualise the Irish situation against international comparators and work across countries to tackle the global challenges of population ageing.”

Is the rationale for developing the new method (or application) clearly explained?

Yes

Is the description of the method technically sound?

Yes

Are the conclusions about the method and its performance adequately supported by the findings presented in the article?

Yes

If any results are presented, are all the source data underlying the results available to ensure full reproducibility?

Partly

Are sufficient details provided to allow replication of the method development and its use by others?

Yes

Reviewer Expertise:

Microsimulation and health/ageing/social services

I confirm that I have read this submission and believe that I have an appropriate level of expertise to confirm that it is of an acceptable scientific standard, however I have significant reservations, as outlined above.

# Response

**Published:** 2022-05-30

**Authors:** Peter May

**Affiliations:** Trinity College Dublin, Ireland

Thanks to the reviewer for their detailed comments. We have numbered (R1.1, R1.2, etc) and responded to specific comments below marked *>, and we have revised the manuscript accordingly. The paper is now stronger as a result. Where responses are relevant to Reviewer 2 comments, we have flagged the relevant response (R2.x).

Methods

 R1.1 It is unclear whether care homes are included in the model.

 *>With respect to sampling, we have clarified in Methods>Data:

 “While the cohort was recruited to be representative of community-dwelling people aged over 50 at baseline, once participants move into residential care they remain part of the study, and so over time the composition of the sample has become more representative of the entire population aged over 50.”

 *>With respect to care homes as an outcome variable, we have clarified in Discussion>Future iterations of the model:

 “TILDA collects usage across more than 20 categories of health care use, including time spent in a nursing home.”

 R1.2 What proportion of the population has private health insurance?

 *>Approximately one-third of the sample have neither public nor private insurance cover. These data have been published previously (Table 9.4 in https://tilda.tcd.ie/publications/reports/pdf/w4-key-findings-report/TILDA-Wave4-Key-Findings-report.pdf); we don’t report the data in our paper since insurance cover is not a predictor in our model. We will investigate the usefulness of including this as an independent variable in future iterations.

 R1.3 Are carers included in the data?

 *>Clarification in Discussion>Planned future iterations:

 “household economic outcomes: *unpaid care*, income, pension and assets. These are beyond the scope of our current IFOAM project but we have the possibility to extend to these outcomes in future.”

 R1.4 What is the retention rate for the survey?

 *>These data are provided in Results>Mortality and attrition, and in Table 4.

 R1.5 Was the mortality data linked to the survey underpinning the model - if not, what was it used for?

 *>This is stated in Methods>Dependent variables:

 “Mortality was identified through GRO linkage”.

 R1.6 Did the data include only the six health conditions/risk factors referred to in Table 1 or was this a subset used for the model?

 *>Clarification added to Methods>Dependent variables:

 “We selected six diagnoses from a longer list of data collected by TILDA.”

 *>Clarification added to Results>Future iterations:

 TILDA collects data on diagnoses that are important for health and health care use but not included in this paper; e.g. serious liver disease, serious kidney disease, Alzheimer’s disease and related dementias.  We will incorporate additional diagnoses in future work as relevant to the specific research questions.

 R1.7 Table 2: BMI is also a risk factor for cancer and stroke; smoking is also a risk factor for heart disease and stroke.

 *>Smoking variables are in the models for heart disease and stroke, and their omission was an error. This has been corrected in Table 2. BMI is an unusual variable in our data due to its high missingness (see also R1.11 below). We looked at model performance for diabetes, cancer, and stroke with and without BMI as a predictor, weighing up the trade-off between the additional predictor and the additional sample size each time. We found that BMI improved model performance in diabetes but did not significantly improve the other models in our data. Our results, in terms of projections and validation, are substantively unaffected either way.

 R1.8 How many times were the scenarios run to establish confidence intervals?

 *>We have amended our description in Methods>Statistical methods, including a brief explanatory appendix document:

 “We managed this uncertainty by rerunning the scenarios 25 times and reporting the mean of these outputs. We do not report confidence intervals from these 25 scenarios since we consider this difficult to interpret: as the number of Monte Carlo simulations increases, the confidence intervals tend towards zero.  See supplementary file ‘Managing uncertainty in IFOAM’ for further details.”

 R1.9 Are there plans to include important trends such as increasing education and declining smoking in the model?

 *>Clarification added in Discussion>Limitations

 “In the model reported here we did not otherwise adjust the replenishing sample characteristics (e.g. to incorporate cohort effects of increased education access, declining tobacco consumption). We will address this in future iterations using data from Healthy Ireland and the Census to model cohort effects in our predictors.”

Results

 R1.10 Diagnoses: Hypertension is typically considered a risk factor rather than a disease.

 *>We have changed the terminology from diagnoses to chronic (health) conditions throughout the paper.

 R1.11 Missing data: The 28% missing BMI data in wave 1 is high - could this be imputed from subsequent waves assuming BMI remained similar over time? This seems important given the importance of BMI as a risk factor.

 *>Imputing BMI at Wave 1 is complicated by a measurement inconsistency across Waves, detailed in the Legend for Table 1: “BMI, which was measured by a nurse in a health assessment centre at Wave 1 and self-reported in all subsequent waves. This created an inconsistency problem, where Wave 1 BMI was higher than at later Waves for the majority of participants. For data consistency we used BMI as an outcome in Wave 2 onwards only, and we used BMI as a predictor in all waves after adjusting the Wave 1 data to match the distribution of later Waves (e.g. if a participant had BMI in the 60 th percentile in Wave 1 then we adjusted their Wave 1 BMI to the 60 th percentile value for later waves).”

 Per R1.7 above, we have performed sensitivity analyses on some regressions to see if including or excluding BMI is affecting results. We have found that it does not.

 R1.12 Missing outcome data: The last sentence "Missing BMI data remained higher Wave 2...." doesn't seem to make sense - is there a word or two missing?

 *>Sentence revised:

 “From Wave 2 onwards, BMI was added to the CAPI and missingness was 6%-12% – that is, much lower than BMI missingness at Wave 1 but notably higher than missingness in any other variable in the models.”

 R1.13 No significance testing was reported - could this be added to comment on the importance of change over time or will this be in a future more detailed analysis paper?

 *>R1.8 above, we don’t think statistical testing is appropriate using the approach in this illustrative methods paper. We do accept the importance of uncertainty in projects and will take more detailed steps to address it when answering specific policy questions.

Discussion

 R1.14 What will the model be used for - projecting expenditure, resource planning, etc.?

 The model seems as though it would be useful for projecting the increasing rate of comorbidity and frailty as the population ages - important in relation to duration and cost of hospital admissions for example.

 *>Added to Discussion>Future iterations:

 “The model can now be used to project the prevalence of individual diseases, overall comorbidity burden and functional limitations, and associated health care use, among older people in Ireland. We can estimate future health problems and expenditures, translate smaller scale intervention effects into population estimates, and evaluate ex ante policy choices designed to improve health and/or the value of care provided to this population.”

 R1.15 If there is a more comprehensive data set with, for example, 20 categories of healthcare use and perhaps many more health conditions why was it not used at the outset?

 *>The total number of diagnoses and health care use categories in TILDA offers a very large number of potential models, and it’s not feasible to model and report all such permutations each time. Therefore we aim first (in this paper) to document the methods used (see also Introduction>Aim). And in future papers, we will tailor the choice of outcomes to specific research questions (see Discussion>Future iterations), where the methods documented here will be an important reference point.

 R1.16 The paper has several particular strengths which I thought were covered quite well in the paper immediately before the Limitations section in the discussion, although not under the specific heading of strengths. Perhaps prefacing the following material with the heading Strengths and creating a new paragraph as follows would suffice:

 *>We appreciate the sentiment but the journal style is to follow STROBE, which has no strengths section, so we are satisfied to leave it as is.
